# Influences of *Dryopteris crassirhizoma* Extract onthe Viability, Growth and Virulence Properties of *Streptococcus* *mutans*

**DOI:** 10.3390/molecules17089231

**Published:** 2012-08-02

**Authors:** Suk-Ho Ban, Jeong-Eun Kim, Santosh Pandit, Jae-Gyu Jeon

**Affiliations:** 1 Department of Preventive Dentistry, School of Dentistry, Institute of Oral Bioscience and BK 21 Program, Chonbuk National University, Jeonju 561-756, Korea; 2 Natural Product Research Group in Oral Biology (NatPROB), Jeonju 561-756, Korea

**Keywords:** Streptococcus mutans, Dryopteris crassirhizoma, virulence properties

## Abstract

*Dryopteris crassirhizoma* is traditionally used as an herbal remedy for various diseases, and has been identified in a previous study as a potential anti-caries agent. In this study, the effect of a methanol extract of *D. crassirhizoma* on the viability, growth and virulence properties of *Streptococcus mutans*, a cariogenic dental pathogen, was investigated. In addition, the phytochemical composition of the extract was analyzed. The extract showed bactericidal and bacteriostatic activity against oral bacteria (MIC and MBC of *S. mutans*: 62.5 and 250 μg/mL, respectively). At two times the MBC, the extract significantly eliminated *S. mutans* up to 99.9% after 1 h incubation. The extract also dose-dependently reduced growth rates of *S. mutans* at sub-MIC levels. Furthermore, at sub-MIC levels, virulence properties (acid production, acid tolerance, glucosyltransferase activity and sucrose-dependent adherence) of *S. mutans* were also inhibited in a dose-dependent manner. GC-MS analysis revealed the presence of mono and disaccharides (44.9%), fatty acids (12.3%) and sugar alcohols (6.8%) in the extract. These data indicate that the extract might be useful for the control of dental caries.

## 1. Introduction

Dental caries is a biofilm-related oral disease, which continues to afflict the majority of the World’s population [1]. The disease results from the interaction of specific bacteria with constituents of the diet within a biofilm formed on the tooth surface clinically known as dental plaque. Although additional microorganisms may be also involved, *Streptococcus mutans* plays a key role in the pathogenesis of the disease. This bacterium is able to: (i) produce and tolerate acids; (ii) synthesize water-insoluble glucan from sucrose through the activity of glucosyltransferases (GTFs); and (iii) adhere tenaciously to acquired pellicle on tooth surfaces [2,3]. The combination of these virulence properties allows *S. mutans* to effectively colonize tooth surfaces and modulate the transition of nonpathogenic to highly cariogenic dental biofilms, which leads to caries formation [4]. Therefore, approaches aimed at inhibiting the viability and virulence properties of *S. mutans* could be precise and selective for the prevention of dental caries.

*Dryopteris crassirhizoma* is a semi-evergreen plant that grows on the deciduous forest floor as a pteridophyte [5]. The plant is widely distributed in Korea, China and Japan, and the roots are traditionally used as an herbal remedy for various diseases, such as tapeworm infestation, the common cold and cancer [6]. Many studies of the plant have revealed numerous pharmacological properties, including anti-oxidant, anti-cancer and anti-bacterial activities [5,6]. Furthermore, a study showed the potential of the plant as an agent for the prevention of oral diseases, such as dental caries [7]. Generally, the pharmacological activities of the plant have been attributed mainly to the presence of triterpene, phloroglucinol, flavonoid and other phenolic analogs [8].

Medicinal plants have been used as a major source of innovative and effective therapeutic agents throughout human history, and have shown promise as a source of components for the development of new drugs [4]. In addition, studies using medicinal plants to prevent or treat oral diseases such as dental caries have received a great deal of attention because the use of commercial chemotherapeutic anti-caries agents, such as chlorhexidine and triclosan, not only remains controversial but also has common side effects including tooth staining and the emergence of bacterial resistance. Recently, several studies have shown the feasibility of using medicinal plants as a source of chemotherapeutic agents for the prevention of oral diseases, particularly dental plaque-related diseases [9,10,11]. A number of compounds, such as epicatechin, allicin and sanguinarine, isolated from medicinal plants, have also been investigated for their efficacy against oral microbial pathogens [12,13].

Despite the potential of *D. crassirhizoma* as an agent for the prevention of dental caries, its effect on the viability and physiological ability of *S. muans* remains unknown. Furthermore, the phytochemical analysis of the plant extract was not performed in the dental field. As part of an ongoing study on the prevention of dental caries by medicinal plants, the purpose of this study was to evaluate the effect of methanol extract of *D. crassirhizoma* on the viability, growth and virulence properties of *S. mutans*, especially its effects on acid production, acid tolerance, GTF activity, and sucrose-dependent adherence. In addition, this study also aimed to investigate the phytochemical composition of the extract.

## 2. Results and Discussion

### 2.1. Phytochemical Composition of the Extract

Metabolite profiling of plant extracts provides information on their phytochemical composition, which allows detection of chemically varied bioactive molecules or unknown compounds. Generally, the two major hyphenated techniques, gas chromatography-mass spectrometry (GC-MS) and liquid chromatography-mass spectrometry (LC-MS), are employed in metabolite profiling of plant extracts [14]. In this study, GC-MS was used for identification of the phytochemical compounds of methanol extract of *D. crassirhizoma* (MED) because of the oily appearance of the extract. Furthermore, the extract was derivatized to analyze the polar compounds in the extract and facilitate chromatographic separation on a column of low polarity [14]. The phytochemical composition of methanol extract of *D. crassirhizoma* was analyzed for the first time by GC-MS in this study.

**Figure 1:**
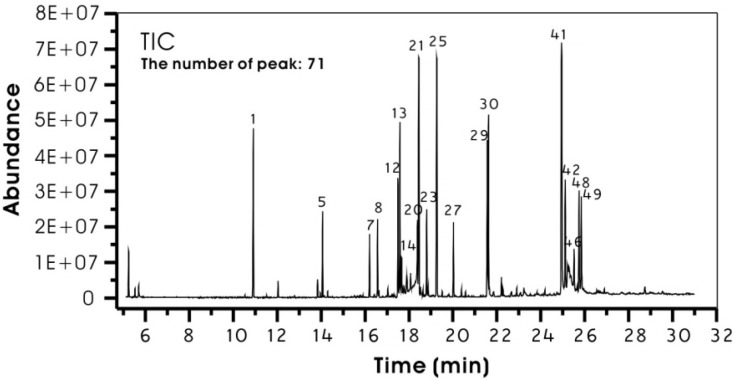
Total ion chromatogram (TIC) of derivertised methanol extract of *Dryopteris crassirhizoma* by GC-MS. Major components were marked on the plot with their peak numbers, which were connected with their chemical names in Table 1.

The results of GC-MS analysis of derivatized MED are shown in Figure 1 and Table 1. About 71 compounds were detected by GC-MS (Figure 1). As shown in Table 1, the principlecompounds were mono- and disaccharides [27.6 and 17.3% of relative percentage of peak area (RAP), respectively], followed by fatty acids (12.3% of RAP) and sugar alcohols (6.8% of RAP). Mono and disaccharides of MED were mainly fructose, glucose and sucrose. Fatty acids of MED were mainly palmitic, linoleic and oleic acids. Interestingly, some sugars (fructose and glucose) generate two peaks. This may be due to their geometric isomers derived from the oxime reaction [15,16]. In general, the identified compounds of MED were mainly primary metabolites, including sugars, fatty acids and sugar alcohols, and total RAP of the compounds was 73.9% (Table 1).

Although previous phytochemical reports on *D. crassirhizoma* revealed the presence of triterpene, phloroglucinol, flavonoid and other phenolic analogs [5], these compounds were not detected in this study (Table 1). This may be because the concentration of the compounds in MED was too low to be detected by the method used in this study. However, since the identified compounds of MED in this study were mainly primary metabolites, further qualitative and quantitative phytochemical studies on MED will be needed to reveal the presence of secondary metabolites, including phenolic compounds and terpenoids.

**Table 1:** Chemical composition of derivertised methanol extract ^a^ of *Dryopteris crassirhizoma*.

Peak No. ^b^	Compounds	Rt ^c^	RAP ^d^ (%)		Peak No. ^b^		Compounds	Rt ^c^	RAP ^d^ (%)
1	Glycerol	10.91	4.57		26		Gluconic acid	19.52	0.16
2	Butenedioic acid	12.04	0.38		27		Palmitic acid	20.03	1.81
3	Malic acid	13.84	0.42		28		Inositol	20.40	0.26
4	Butanal	13.97	0.13		29		Linoleic acid	21.56	4.67
5	Threitol	14.07	2.01		30		Oleic acid	21.62	5.27
6	Proline	14.30	0.17		31		Stearic acid	21.67	0.33
7	U.I	16.21	1.53		32		U.I	21.86	0.14
8	Xylitol	16.58	1.97		33		U.I	22.21	0.53
9	Ribitol	16.63	0.17		34		U.1	22.28	0.20
10	Xylonic acid	17.04	0.29		35		U.I	22.67	0.12
11	Propanoic acid	17.24	0.10		36		Arachidonic acid	22.91	0.25
12	Fructose	17.50	3.14		38		U.I	23.08	0.21
13	Fructose	17.59	4.83		39		U.I	23.24	0.56
14	Citric acid	17.67	1.62		40		U.I	23.83	0.19
15	Benzoic acid	17.72	0.30		41		Sucrose	24.95	11.84
16	U.I	17.79	0.54		42		Lactulose	25.12	3.77
17	U.I	17.89	0.76		43		U.I	25.22	1.42
18	U.I	17.92	0.59		44		U.I	25.28	1.93
19	U.I	18.08	1.55		45		U.I	25.35	0.60
20	U.I	18.39	5.51		46		Maltose	25.52	1.69
21	Glucose	18.45	9.28		47		U.I	25.66	0.31
22	Galactose	18.53	0.46		48		U.I	25.75	3.05
23	Mannitol	18.81	2.26		49		U.I	25.85	3.03
24	Glucitol	18.88	0.37		50		U.I	25.94	0.20
25	Glucose	19.26	9.86			**Total RAP of identified compounds:**			**73.91**

Only the components which have RAP > 0.1% are shown in this table. ^a^ Identified as trimethylsilyl (TMS) derivatives; ^b^ Numbering refers to the order of elution from HP-DB5ms capillary colum; ^c^ Retention time (min); ^d^ Relative percentage of peak area (peak area relative to total area %);U.I: unidentified compounds.

### 2.2. Effect on Bacterial Viability and Growth

In dentistry, when antimicrobial agents are delivered topically by mouth rinse or dentifrice, the agents in the oral cavity may initially be present at a relatively high concentration [≥minimum inhibitory concentration (MIC)], but the concentration of these agents will subsequently be lowered due to expectoration and swallowing [17]. The resulting concentration may be below the MIC. Therefore, in this study, we have examined the antibacterial activity of MED at both higher concentrations (≥MIC) and sub-MIC levels concurrently to determine its potential for the control of dental caries at broad concentrations.

**Figure 2:**
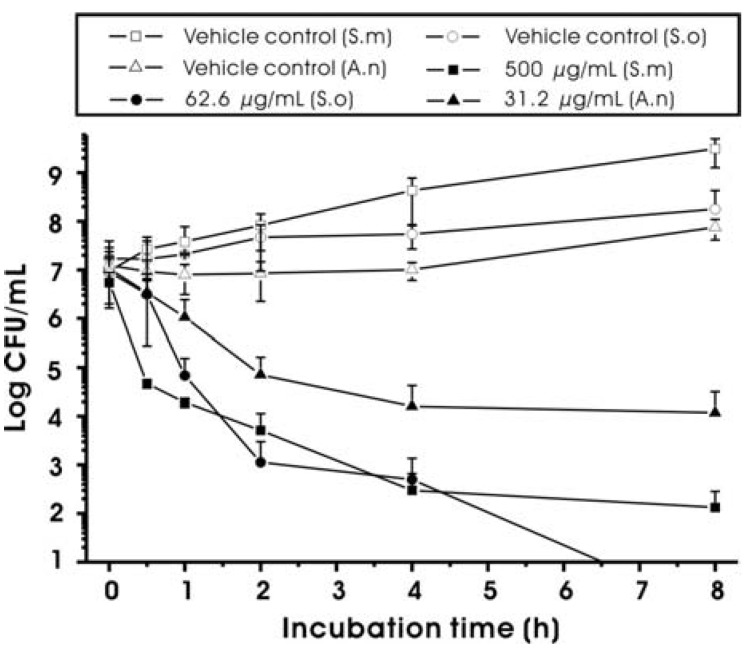
Time-kill curves for *Streptococcus mutans* (S.m), *Streptococcus oralis* (S.o) and *Actinomyces naeslundii* (A.n) in the presence of methanol extract of *Dryopteris crassirhizoma* root (MED). The final concentrations of MED were two times of MBCs against the bacterial species tested. The vehicle controls were 1% DMSO (v/v, final concentration). Data represent mean ± standard deviation.

**Figure 3:**
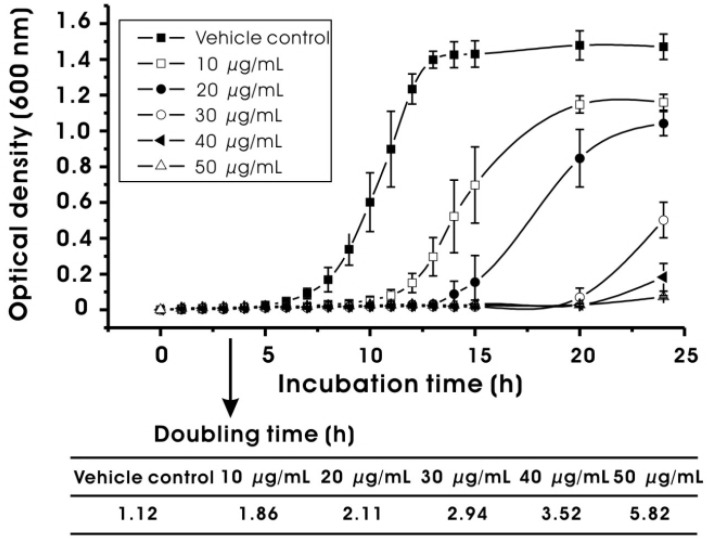
Effect of methanol extract of *Dryopteris crassirhizoma* root (MED) on the growth rate of *Streptococcus mutans*. Growth rate was expressed by measuring doubling time (min) in the presence or absence of MED at sub-MIC levels. The vehicle control was 1% DMSO (v/v, final concentration). Data represent mean ± standard deviation.

The antimicrobial activity of MED against oral bacteria, including *S. mutans*, is shown in Table 2, and Figure 2 and Figure 3. In general, MED showed bacteriostatic or bactericidal activity at higher concentrations (≥MIC) and inhibitory effects on the growth rates of *S. mutans* at sub-MIC levels. As shown in Table 2, the MIC value against *S. mutans* was 62.5 μg/mL. The MIC values for *A. naeslundii* and *S. oralis* were 7.8 and 15.6 μg/mL, respectively, suggesting that the antimicrobial activity is not specific to the cariogenic bacteria and that the MIC values of MED depend strongly on bacterial strains. The minimum bactericidal concentration (MBC) values were two to four times higher than the MIC values (Table 2). The MBC value against *S. mutans* was 250 μg/mL. In order to determine the antimicrobial activity of MED at concentrations higher than MBC, a time-kill assay was performed. As shown in Figure 2, MED at two times the MBC significantly reduced the viable counts of the bacteria tested after 1 h of incubation (*p* < 0.05). Especially, MED produced a 3 log decrease (99.9%) of *S. mutans* after 1 h of incubation. In addition to the bacteriostatic and bactericidal effects at higher concentrations (≥MIC), MED also reduced the growth rates of *S. mutans* at lower concentrations (<MIC). As shown in Figure 3, the doubling time of *S. mutans* at the highest sub-MIC (50 μg/mL) increased up to 5.82 h, while that in the vehicle control were 1.12 h. The level of growth rate inhibition was dependent on MED concentrations.

**Table 2:** Minimum inhibitory concentration (MIC) and minimum bactericidal concentration (MBC) of methanol extracts from *Dryopteris crassirhizoma* root.

Test bacteria	MIC (μg/mL)	MBC (μg/mL)
*Streptococcus mutans* UA159	62.5	250
*Streptococcus oralis* 35037	15.6	31.3
*Actinomyces naeslundii* 12104	7.8	15.6

Vehicle control (1% DMSO, v/v, final concentration) did not show inhibitory effect on the growth of the bacteria tested.

In this study, the antimicrobial activity of MED against oral bacteria was less potent than that of chlorhexidine dihydrochloride (MICs against oral bacteria, including *S. mutans*: 6.25 to 25 μg/mL), which is a clinically proven antimicrobial. This result might be because MED is not purified for the antimicrobial components. Nevertheless, it is meaningful that the MICs of MED were below the concentration of 100 μg/mL, because medicinal plants that exhibit antimicrobial activity at concentrations lower than 100 μg/mL could have great antimicrobial potential, since the active compounds can be isolated and used at lower concentrations [18]. However, it is significant that although there are differences in experimental methods, the antimicrobial activity of MED against oral bacteria was stronger than that of the extracts from other medicinal plants, such as *Camellia sinensis* and *Rosmarinus officinalis* [19], suggesting that if antimicrobial components are isolated from MED, the antimicrobial activity might be clinically useful for the prevention of dental caries. Furthermore, MED caused a rapid decrease in *S. mutans* viable counts at high concentrations and in *S. mutans* growth rates at low concentrations: These findings suggest that MED can be maintained in effective concentrations for a long time in the oral cavity, as delivered by mouth rinse or dentifrice. 

### 2.3. Effect on Virulence Properties of *S. mutans* at Sub-MIC Levels

Among several measures to prevent dental caries, chemotherapeutic approaches could be precise and selective. Chemotherapeutic agents that have biological activities against *S. mutans* would be potential candidates to be used as an adjunct to prevent or reduce dental caries. These agents may prevent dental caries through one or more of the following mechanisms [4]: (1) antimicrobial approaches, which include inhibition of bacterial growth through bactericidal or bacteriostatic mechanisms; and (2) physiological approaches, which involve inhibition of bacterial virulence properties, such as inhibition of bacterial enzymes, gene expression, or proton permeability. In this study, the influence of MED on virulence properties of *S. mutans* at sub-MIC levels was also investigated to clarify the effect on the physiological ability of the bacterium.

**Figure 4:**
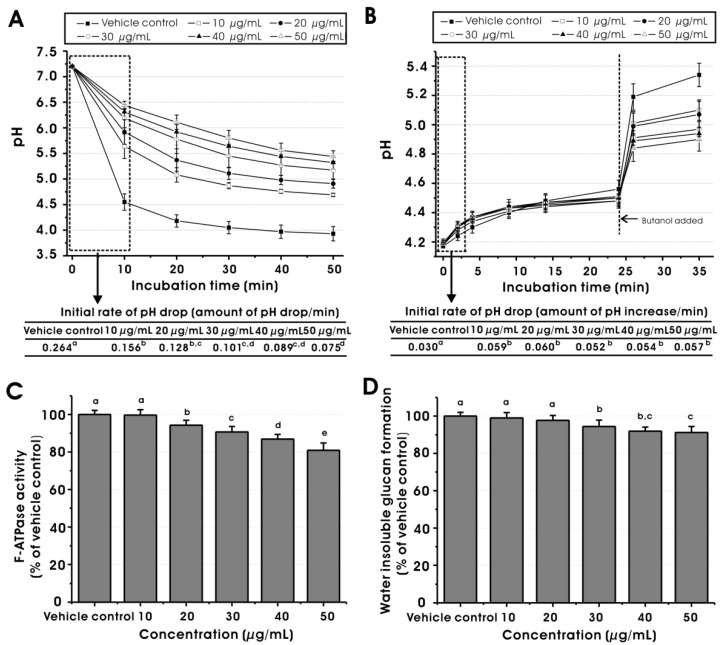
Effects of methanol extract of *Dryopteris crassirhizoma* root on (**A**) glycolytic pH-drop; (**B**) proton-permeability; (**C**) F-ATPase activity and (**D**) GTF activity of *Streptococcus mutans* at sub-MIC levels. The data represent mean ± standard deviation. The vehicle control was 1% DMSO. Values followed by the same superscripts are not significantly different from each other (*p* > 0.05).

Acid production and acid tolerance are important virulence properties of *S. mutans* [20]. As shown in Figure 4A, MED reduced the initial rate of the pH drop of *S. mutans* cells at 10, 20, 30, 40 and 50 μg/mL, compared to the vehicle control (*p* < 0.05), suggesting that the glycolytic acid production of *S. mutans* is inhibited dose-dependently by MED at sub-MIC levels. The inhibitory activity may result from the effect of MED on the bacterial glycolytic pathways because resting bacteria were used for this assay, which means specific inhibition of the glycolytic activity of the bacterial cells rather than inhibition by a decrease in bacterial growth. In addition, Figure 4A shows that the final pH values at 10, 20, 30, 40 and 50 μg/mL (at 50 min incubation) significantly differed from that of the vehicle control (*p* < 0.05), suggesting the disruption of acid tolerance ability of the bacterial cells since final pH values in glycolytic pH-drop assay reflect acid tolerance [21].

In this study, proton-permeability and F-ATPase activity assays were performed to confirm the effect of MED at sub-MIC levels on the acid tolerance of *S. mutans* cells. It is clearly reported that the protons from the extracellular environment diffuse inward across the cell membrane after acidification of the extracellular environment but can then be extruded by the membrane associated F-ATPase enzyme [22]. The F-ATPase protects *S. mutans* against environmental acid stress by regulating pH homeostasis. The result of the proton permeability assay is shown in Figure 4B. MED at 10, 20, 30, 40 and 50 μg/mL increased the permeability of *S. mutans* cells to protons during 4 min incubation periods, compared to the vehicle control (*p* < 0.05). Furthermore, MED reduced the initial rate of proton entry, compared to the vehicle control (*p* < 0.05). However, there was no significant difference in the effect on proton permeability among the concentrations tested. This result may be explained if the response of proton permeability by the application of MED is all or nothing, or the response reaches the maximum under 10 μg/mL. The result of the F-ATPase activity assay is shown in Figure 4C. The F-ATPase activity of *S. mutans* cells was inhibited dose-dependently at 20, 30, 40 and 50 μg/mL, compared to the vehicle control (*p* < 0.05). Overall, it is apparent that the inhibitory effect of MED on the acid tolerance of *S. mutans* cells is related, in part, to a disruption of the proton permeability of the membrane and F-ATPase activity, which would affect the pH gradient across the membrane, leading to an inhibition of the overall intracellular metabolism of the bacterial cells.

**Figure 5:**
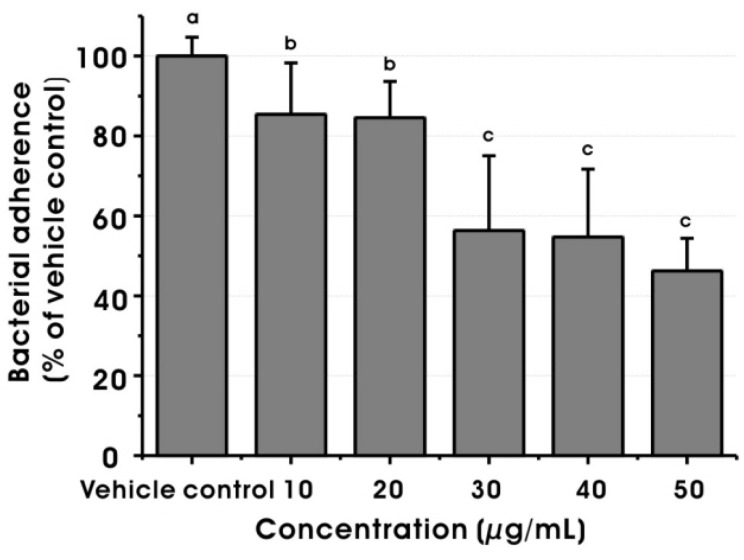
Effect of methanol extract of *Dryopteris crassirhizoma* root on sucrose-dependent adherence of *Streptococcus mutans* at sub-MIC levels. The data represent mean ± standard deviation. The vehicle control was 1% DMSO. Values followed by the same superscripts are not significantly different from each other (*p* > 0.05).

It is well known that GTFs of *S. mutans* produce water-insoluble glucans from sucrose to contribute bacterial adherence and formation of dental biofilms [23]. Thus, water-insoluble glucan formation by GTFs is one of the most important caries-inducing factors of *S. mutans* that deserves attention during an examination of medicinal plants for the prevention of dental caries. As shown in Figure 4D, MED significantly reduced water-insoluble glucan formation by crude GTFs from *S. mutans* at 30, 40 and 50 μg/mL (*p* < 0.05). Furthermore, MED significantly decreased the sucrose-dependent adherence of *S. mutans* to hydroxyapatite discs at 10, 20, 30, 40 and 50 μg/mL in a dose-dependent manner (Figure 5). Based on these results, it is apparent that MED could inhibit the GTF activity of *S. mutans* and thereby sucrose-dependent bacterial adherence, suggesting that the agent may modulate the development and accumulation of dental biofilms.

Although the precise bioactive compounds of MED that induce the inhibitory effects on the viability and virulence properties of *S. mutans* are not revealed in this study, the pharmacological activities of the extract might be related to the presence of fatty acids, especially linoleic and oleic acid. There are a number of studies concerning the antimicrobial effects of various fatty acids, including linoleic and oleic acid, from a wide range of biological sources, such as algae, animals and plants [24]. In addition, a previous study reported that linoleic and oleic acids from cacao bean husks show strong antimicrobial activity against *S. mutans* [25]. Furthermore, fatty acids are considered as potent inhibitors of diverse bacterial enzymes in the membrane or cytosol [24,26]. Thus, it is possible to suppose that fatty acids in MED can reduce the enzyme activity of *S. mutans*, such as glycolysis enzymes, proton-translocating ATPase and GTFs, thereby leading to the decrease of the acid production and tolerance, and water-insoluble glucan formation by the bacterium at lower concentrations than MIC. At higher concentrations of MED, the damage could be too great to repair, leading to the cell death or growth inhibition of the bacterium. However, future studies will need to determine the precise bioactive compounds of MED that can show the inhibitory effects against *S. mutans.*

## 3. Experimental

### 3.1. Bacterial Strains and Culture Medium

*S. mutans* UA159 (serotype c), a proven cariogenic dental pathogen, was used mainly in this study. For MIC and MBC determination, and time-kill assay, *Actinomyces naeslundii* 12,104 and *Streptococcus oralis* 35,037 were used as well as *S. mutans*. All the bacterial strains were grown in ultrafiltered tryptone-yeast extract (UTE) broth with 1% glucose at 37 °C under 5% CO_2_.

### 3.2. Plant Extraction and Chemical Characterization by GC-MS

The root of *D. crassirhizoma* was purchased from an herbal drug market (Jeonju, Korea). A voucher specimen (PD0504) has been deposited at the Institute of Oral Bioscience, Chonbuk National University. The dried and finely ground plant material (100 g) was macerated with 99.9% methanol (1 L) at room temperature for 24 h. The extract was filtered, concentrated under reduced pressure at a temperature lower than 40 °C, and lyophilized, resulting in 13.9 g methanol extract of *D. crassirhizoma* (MED). MED was maintained at −20 °C until tested. 

For the evaluation of the phytochemical composition of MED, GC-MS analysis was carried out on an Agilent HP-6890 GC/5973 mass selective detector (Agilent Technologies, Palo Alto, CA, USA); a HP-DB5ms capillary column (30 m × 0.25 mm × 0.25 µm) was used. The sample was dissolved in 100 μL of pyridine and derivatised for 30 min at 70 °C with *N,O*-bis(trimethylsilyl)trifluoroacetamide (BSTFA) + 1% trimethylchlorosilane (TMCA). One μL of the derivatised extract was injected. The injector port was heated to 280 °C. Injection was performed in split mode, with a ratio of 1/30. The carrier gas was helium at a constant flow of 1 mL/min. The oven temperature was initially set at 70 °C for 3 min, then increased to 10 °C/min to 300 °C and held for 5 min. Spectra were obtained in the EI mode with 70 eV ionization energy. The sector mass analyzer was set to scan from 50 to 600 amu. The identification of compounds was achieved by comparison of their mass spectra with those in the mass spectra library [27].

### 3.3. Effect on Bacterial Viability and Growth

#### 3.3.1. Determination of MIC and MBC

The MIC of MED was determined using a micro dilution method, as described by Song *et al*. [28]. Inoculum suspensions were prepared from 18 to 24 h broth cultures. Diluted 100 μL suspensions of each bacterial strain were added to 100 μL of various concentrations of MED diluted with UTE broth to obtain a final bacterial count of approximately 1–1.5 × 10^6^ CFU/mL. The final concentrations of MED ranged from 250 μg/mL to 3.9 μg/mL in a series of two fold dilutions. The MIC was defined as the lowest concentration that restricted bacterial growth to an absorbance lower than 0.05 at 550 nm. For the determination of the MBC, an aliquot (100 μL) of all the wells with concentrations higher than the MIC was removed, diluted 10-fold in 0.85% saline, and then sub-cultured on Brain Heart Infusion (BHI) (Difco, USA) agar plates. The MBC was defined as the lowest concentration that did not permit any visible growth on the agar plate after 48 h incubation.

#### 3.3.2. Time-kill Assay

Time-kill assay was performed according to the method of Song *et al*. [28]. The starting inoculum was 5 × 10^6^ to 2 × 10^6^ CFU/mL. The final concentration of MED was two times the MBC. Tubes containing the bacterial species, and MED or the vehicle control [1% dimethyl sulfoxide (DMSO)] in UTE broth were incubated at 37 °C. Samples were removed for determination of viable counts at 0 and 30 min and 1, 2, 4, and 8 h. Serial dilutions (10^−1^ to 10^−4^) were prepared in 0.85% saline. The diluted sample (100 μL) was plated onto BHI agar plates. The plates were incubated at 37 °C for 48 h, and the number of colonies was determined.

#### 3.3.3. Bacterial Growth Rate at Sub-MIC Levels

In order to examine the effect of MED on the growth rate of *S. mutans* at sub-MIC levels, the doubling time of bacterial growth was determined. For this assay, *S. mutans* was grown at 37 °C in UTE broth containing sub-MIC levels of MED (10–50 μg/mL) or the vehicle control (1% DMSO). The bacterial growth was monitored spectrophotometrically at 550 nm against a culture medium blank, at selected time intervals. The doubling time of the bacterial growth was calculated by measuring the slope of the logarithmic growth phase according to the method of Khalichi *et al*. [29].

### 3.4. Effect on Virulence Properties of *S. Mutans* at Sub-MIC Levels

#### 3.4.1. Glycolytic pH-drop Assay

The level of the glycolytic pH drop of *S. mutans* was measured, as described elsewhere [30]. Briefly, the cells of *S. mutans* from the suspension cultures were harvested, washed once with salt solution (50 mM KCl + 1 mM MgCl_2_) and resuspended in a salt solution containing MED (final concentration: 10–50 μg/mL) or the vehicle control (final concentration: 1% DMSO). The pH was adjusted to 7.2–7.4 with 0.2 M KOH solution. Sufficient glucose was then added to obtain a concentration of 1% (w/v) and the decrease in pH was assessed over a period of 50 min using a glass electrode (Futura Micro Combination pH electrode, 5 mm diameter, Beckman Coulter Inc., Brea, CA, USA). The initial rate of the pH drop, which can give the best measure of the acid production capacity of the cells, was calculated using the pH values in the linear portion (0–10 min).

#### 3.4.2. Proton Permeability Assay

The proton permeability of *S. mutans* was assessed using standard procedures described by Phan *et al*. [31]. Briefly, the cells of *S. mutans* from the suspension cultures were initially washed with salt solution (50 mM KCl + 1 mM MgCl_2_). Subsequently, they were incubated in salt solution at a constant pH value of approximately 4.6. Then, HCl was added to drop pH values by about 0.4 units followed by the addition of MED (final concentration: 10–50 μg/mL) or the vehicle control (final concentration: 1% DMSO). The subsequent rise in pH associated with the movements of protons across the cell membrane into the cytoplasm was monitored with a glass electrode. The initial rate of proton entry was also estimated because changes in initial rates of proton uptake give the best measure of disruptive effects. The proton entry rate was calculated using the pH changes of a 0–2 min incubation period. Butanol (final concentration: 10%) was added to the suspensions at 25 min to damage the cell membrane.

#### 3.4.3. F-ATPase Activity Assay

F-ATPase activity assay was performed using permeabilized cells of *S. mutans* by subjecting the cells to 10% toluene (v/v) followed by two cycles of freezing and thawing as described elsewhere [30]. F-ATPase was measured in terms of the release of phosphate in the following reaction mixture: 75 mmol of Tris-maleate buffer (pH 7.0) containing 5 mM ATP, 10 mmol of MgCl_2_, permeabilized cells, and MED (final concentration: 10–50 μg/mL) or the vehicle control (final concentration: 1% DMSO). The released phosphate (over the 10 min reaction time) was determined by the method of Bencini *et al*. [32].

#### 3.4.4. GTF Preparation and GTF Activity Assay

The level of water-insoluble EPS formation by crude GTFs was measured. The crude GTFs of *S. mutans* were precipitated from the culture supernatant by adding solid ammonium sulphate and were then recovered, as detailed elsewhere [33]. The reaction mixture (total volume 1 mL) consisted of the following: (a) 300 μL of the enzyme; (b) 175 μL of 0.01 M potassium phosphate buffer (pH 6.8) containing NaN_3_ (3 mM); (c) 500 μL of 0.01 M potassium phosphate buffer (pH 6.8) containing 5% sucrose and NaN_3_ (3 mM); and (d) MED (final concentration: 10–50 μg/mL) or the vehicle control (final concentration: 1% DMSO). NaN_3_ was added to prevent contamination by microorganisms. The reaction time was 24 h.

#### 3.4.5. Sucrose-dependent Adherence Assay

The effect of MED at sub-MIC levels on the sucrose-dependant bacterial adherence was determined by counting the number of colony forming units (CFUs) after treatment [33]. For the adherence assay, saliva-coated hydroxyapatite discs were treated with MED (final concentration: 10–50 μg/mL) or the vehicle control (final concentration: 1% DMSO) for 10 min. After treatment, the discs were dip-rinsed three times in autoclaved water (to remove the excess agents or the vehicle control) and immersed in 1% sucrose UTE broth containing *S. mutans* UA159 (2–3 × 10^6^ CFU/mL). The discs were incubated for 2 h at 37 °C with 5% CO_2_. After incubation, the bacteria were collected by sonication and plated onto BHI agar to count the number of colonies. 

### 3.5. Statistical Analyses

The data are presented as mean ± standard deviation. The intergroup differences were estimated by one-way analysis of variance (ANOVA), followed by a post hoc multiple comparison (Tukey test) to compare the multiple means. Values were considered statistically significant when *p* value was <0.05. The statistical analyses were performed using SPSS 12 software (SPSS Inc., Chicago, IL, USA).

## 4. Conclusions

In conclusion, the results of the present study showed that methanol extract of *D. crassirhizoma*, mainly composed of mono- and disaccharides, fatty acids and sugar alcohols, has bactericidal and bacteriostatic activity at high concentrations (≥MIC) and inhibitory effects on the acid production, acid tolerance, water-insoluble glucan formation and sucrose-dependent adherence of S. *mutans* at sub-MIC levels, suggesting that it might be useful for control of *S. mutans*, a primary etiologic agent of dental caries, and its biofilm formation. However, further phytochemical investigations will be needed to isolate the active compounds, such as palmitic, linoleic and oleic acids 
